# A comparative study on selectivity and sensitivity of new chromium and copper electrodes

**DOI:** 10.1038/s41598-022-17662-6

**Published:** 2022-08-04

**Authors:** Elnaz Bakhshi Sarabi, Leila Hajiaghababaei, Mohammad Reza Allahgholi Ghasri, Seyed Enayatollah Mottaghinejad, Ali Parsa

**Affiliations:** Department of Chemistry, Yadegar-e-Imam Khomeini (RAH) Shahre Rey Branch, Islamic Azad University, Tehran, Iran

**Keywords:** Environmental sciences, Chemistry, Materials science

## Abstract

4-Methylcoumarin-7-yloxy-N-phenyl acetamide and 4-methylcoumarin-7-yloxy-N-4-nitrophenyl acetamide were synthesized and used as new ionophores in the carbon paste matrix to produce two novel potentiometric modified electrodes. The selectivity of the electrode changed from copper (II) to chromium (III) with the addition of a nitro group to the phenyl ring of the ionophore. The ionophores’ tendency to ions was confirmed by UV–visible spectrophotometry. Both electrodes were modified by multi-walled carbon nanotubes (MWCNTs) as an excellent modifier of carbon paste electrode (CPE). The best sensor response in the case of copper (II) selective CPE was obtained by 5% ionophore, 65% graphite powder, 5% MWCNT, and 25% paraffin oil. In addition, in the case of chromium (III) selective CPE, these conditions are 20% ionophore, 50% graphite powder, 5% MWCNT, and 25% paraffin oil. The copper (II) selective CPE showed a Nernstian slope of 32.15 mV/decade within the concentration range of 1.0 × 10^–10^–1.0 × 10^–1^ mol L^−1^, while chromium (III) selective CPE showed a Nernstian slope of 19.28 mV/decade over the concentration range of 1.0 × 10^–10^–7.0 × 10^–3^ mol L^−1^. The electrodes have short response time of less than 5 s and were used successfully to determine copper (II) in wastewater and to speciation of chromium (III) and chromium (VI).

## Introduction

Toxic metals have been discharged into the environment because of global industrialization and have caused serious concerns worldwide by affecting human health^1,2^. For example, copper is needed for the appropriate function of several important enzyme systems. Enzymes containing copper are cytochrome-c oxidase, ceruloplasmin, monoamine oxidase, tyrosinase, phenylalanine hydroxylase, and lysyl oxidase. On the other hand, toxicity is caused by the excess quantity of copper. For instance, Wilson disease is an autosomal recessive disorder caused by copper accumulation in the eyes, liver, and brain. Wilson disease influences the hepatic intracellular transport of copper and its consequent inclusion into bile and ceruloplasmin^3^. In addition, two various oxidation states of chromium normally found in natural waters are Cr (III) and Cr (VI). Both chromium types enter the environment from different sources at the effluent discharge of electroplating, tanning industries, cooling water power, oxidative dying, steelworks, and chemical industry^4^. Chromium has totally opposite physiological effects on the biological systems regarding its oxidation state. Chromium (III) is a key element in mammals to maintain lipid, glucose, and protein metabolism. However, Cr (VI) is a toxic material as it can oxidize other species and has adverse impacts on the lung, kidneys, and liver. Thus, it is essential to determine both species considering these two contrary impacts precisely. Several speciation studies have been conducted on different features and toxicity of the chemical forms of chromium^4–6^. Therefore, it is a critical issue to measure these ions owing to the narrow boundary between their toxicity and essentially.

Analytical methods such as atomic absorption spectroscopy (AAS)^7–9^, X-ray fluorescence (XRF)^10,11^, and inductively coupled plasma-atomic emission spectroscopy (ICP-AES)^12–14^ are performed to determine the trace level of metals like copper and chromium. Nevertheless, these procedures have some disadvantages based on the cost and time of routine analysis and the tedious sample preparation process. Hence, the search for new simple and rapid methods for heavy metal measurement is a challenging goal.

Electrochemical sensors are extensively used for determining different species regarding their desirable selectivity and higher sensitivity in their responses^15–28^. In this regard, potentiometric carbon paste electrodes (CPEs) are extensively used as simple instruments for detecting different analytes regarding their facile construction, higher signal-to-noise ratio, easy renewability of the surface, stability of response, low-cost methods, lower ohmic resistance, and not requiring the internal solution^29,30^. More prominently, carbon paste electrodes can be simply modified with different kinds of inorganic or organic modifiers by only mixing the modifier with binder and carbon in the paste preparing stage. The electrode’s surface state can be improved by modification, which significantly increases the target signals. The potentiometric CPEs’ ion-sensing features are primarily based on the utilized sensing materials’ nature^25–32^. Recently, carbon-based nanomaterials have been widely exploited as electron–ion exchangers to improve electrode performance because of their higher specific surface area, good hydrophobicity, chemical stability, and electrical conductivity^16–29^.

In this research, we synthesized 4-methylcoumarin-7-yloxy-N-phenyl acetamide (ligand A) and 4-methylcoumarin-7-yloxy-N-4-nitrophenyl acetamide (ligand B) for the first time and used them as new ionophores to fabricate modified carbon paste electrodes for measurement of the copper (II) and chromium (III) ions potentiometrically. These two ionophores have a slight difference in structure, which is related to the existence of a nitro group in the phenolic ring of one of them. Therefore, the effect of ionophore molecular structure on selectivity was investigated, and affinity to several metals was compared. Also, both electrodes were modified by multi-walled carbon nanotubes (MWCNTs) and found that the electrodes’ response was improved using MWCNTs. To the best of our knowledge, these are the first ion selective CPEs, prepared with these ionophores as the recognition element of the electrode.

## Results and discussion

In this study, after synthesis of the ligand A and B, their interaction with some metal cations was evaluated by UV–visible spectrophotometry. The reason for this evaluation was to find the suitable cation to form the most stable complex with each ligand. Then, two new modified carbon paste electrodes were fabricated for the potentiometric measurement of copper (II) and chromium (III). Composition of the modified CPEs was optimized, and the effect of various parameters on the potential response of two modified electrodes was studied. The results of these investigations are presented below.

### Evaluation of the ionophores affinity

**Figure 1:**
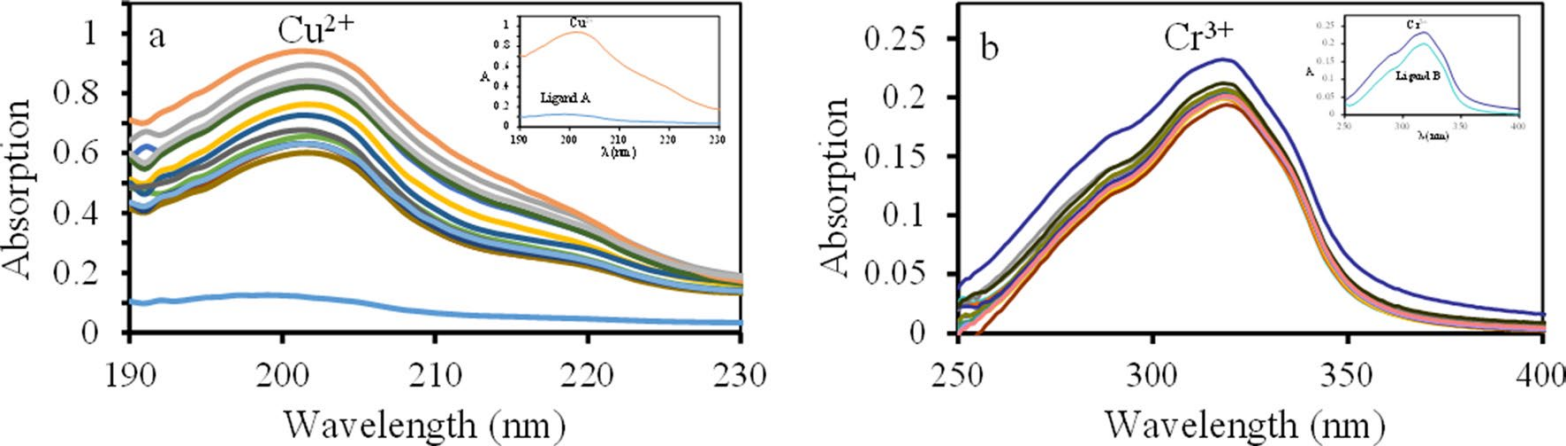
The UV–visible spectrum changes of 1.0 × 10^–5^ M (**a**) ligand A and (**b**) ligand B solution, after addition of 300 µL of 1 mM solutions containing one of the Cr^3+^, Cu^2+^, Ca^2+^, Co^2+^, Mg^2+^, Fe^2+^, Zn^2+^, Ag^+^, Mn^2+^, Hg^2+^, Cd^2+^, Ni^2+^ and Pb^2+^ cations.

Achieving the high selectivity of the potentiometric sensor depends on discovering the suitable cation to form the most strong and stable complex with the sensing material. Because ligand A and B have electron-donating atoms (nitrogen and oxygen) in their structure, their complexation with transition or soft metals is predictable. The ionophore’s affinity to several metals was scrutinized by UV–visible spectrophotometry before preparing the modified CPEs^21,33^. Therefore, equivalent quantities of different ions (Cr^3+^, Cu^2+^, Ca^2+^, Co^2+^, Mg^2+^, Fe^2+^, Zn^2+^, Ag^+^, Mn^2+^, Hg^2+^, Cd^2+^, Ni^2+^ and Pb^2+^) were added to each ionophore solution one by one. The UV–Vis spectra (Fig. 1) were recorded to assess the absorption alterations.

The ligand A and B spectra showed the highest variation in the ionophore absorption after copper (II) and chromium (III) addition (Fig. 1a,b). This result can be attributed to the more affinity for complexing between the ligand A and copper (II), and complexing between ligand B and chromium (III) compared to the other ions. Hence, 4-methylcoumarin-7-yloxy-N-phenyl acetamide and 4-methylcoumarin-7-yloxy-N-4-nitrophenyl acetamide can serve as neutral ion carriers in producing copper (II) and chromium (III) modified CPEs, respectively.

### Electrode composition and modification

The linear dynamic range, sensitivity, and selectivity of an ion-selective electrode are a function of its composition^29^. Therefore, the effect of the paste composition on the modified CPEs’ potential responses was investigated. To this end, several electrodes were fabricated with various compositions (Table 1). Unmodified CPE was made by combining 75% of graphite powder with 25% of paraffin (no. 1). Based on the obtained results, the electrode without the ionophore represented poor sensitivity to copper (II) and chromium (III) cations. Modified pastes were made similarly. However, the graphite powder was combined with a favored weight of ligand A or B, as given in Table 1. As can be seen, an increase in ligand A level in the CPEs (no. 2 and 3) from 3 to 5 mg led to an increased slope of the calibration curve for copper (II) selective electrode from 19.72 mV/decade to 25.45 mV/decade. Also, increasing the amount of the ligand B in the CPEs (no. 7–9) from 5 to 20 mg led to an increased slope of the calibration curve chromium (III) selective electrode from 12.07 mV/decade to 15.11 mV/decade. It was revealed that the quantity of the ionophore is the key factor in the suggested carbon paste electrodes with the main role in the potential response of the sensors. Improving the response of electrodes by increasing the ionophores amounts shows the affinity of the ligand A and B to copper (II) and chromium (III) ions, respectively^29^. But, more increase in the ligand A (no. 4 and 5) lowered the calibration curve slope for copper (II) selective electrode, which can be due to heterogeneity in the carbon paste.

**Table 1:** Optimizing the carbon paste composition.

No.	Paraffin	Graphite	MWCNT_S_	Ligand (A)*	Ligand (B)**	Slope Cu^2+^ ISE (mV/decade) (working range, M)	Slope Cr^3+^ ISE (mV/decade) (working range, M)
1	25	75	0	0	0	13.55 (1 × 10^–9^–1 × 10^–1^)	8.92 (1 × 10^–9^–1 × 10^–3^)
2	25	72	0	3	–	19.72 (1 × 10^–8^–1 × 10^–1^)	–
3	25	70	0	5	–	25.45 (1 × 10^–8^–1 × 10^–1^)	–
4	25	65	0	10	–	20.18 (1 × 10^–8^–1 × 10^–1^)	–
5	25	60	0	15	–	21.69 (1 × 10^–6^–1 × 10^–1^)	–
6	25	65	5	5	–	32.15 (1 × 10^–10^–1 × 10^–1^)	–
7	25	70	0	–	5	–	12.07 (1 × 10^–8^–1 × 10^–3^)
8	25	65	0	–	10	–	13.88 (1 × 10^–8^–1 × 10^–3^)
9	25	55	0	–	20	–	15.11 (1 × 10^–8^–1 × 10^–3^)
10	25	50	5	–	20	–	19.28 (1 × 10^–10^–7 × 10^–3^)

*A: 4-methylcoumarin-7-yloxy-N-phenyl acetamide.

**B: 4-methylcoumarin-7-yloxy-N-4-nitrophenyl acetamide.

The MWCNTs can be used as a superior candidate for the conventional modifier to modify CPE and, thus, improve the electrodes’ electrochemical performance. The main reason for using this structure is its greater electrical conducting capability and higher specific surface area^30^. Hence, we also assessed the effects of inserting some MWCNTs in the electrode composition on the CPEs’ potentiometric response. According to Table 1, both electrodes show the Nernstian slope when MWCNTs were used in the carbon paste composition (no. 6 and 10). Also, the addition of MWCNTs in the electrode composition improves the detection range of the electrode from 1 × 10^–8^–1 × 10^–1^ mol L^−1^ to 1 × 10^–10^–1 × 10^–1^ mol L^−1^ for copper (II) electrode (no. 3 and 6) and from 1 × 10^–8^–1 × 10^–3^ mol L^−1^ to 1 × 10^–10^–7 × 10^–3^ mol L^−1^ for chromium (III) electrode (no. 9 and 10).

Accordingly, a composition of 5% of ligand A, 65% graphite powder, 5% MWCNT, and 25% paraffin oil was found to have the best performance and was chosen as the optimal composition for copper (II) electrode. Furthermore, a composition of 20% ligand B, 50% graphite powder, 5% MWCNT, and 25% paraffin oil showed the best performance and was chosen as the optimal composition for chromium (III) electrode.

### Effects of pH on the modified CPE response

**Figure 2:**
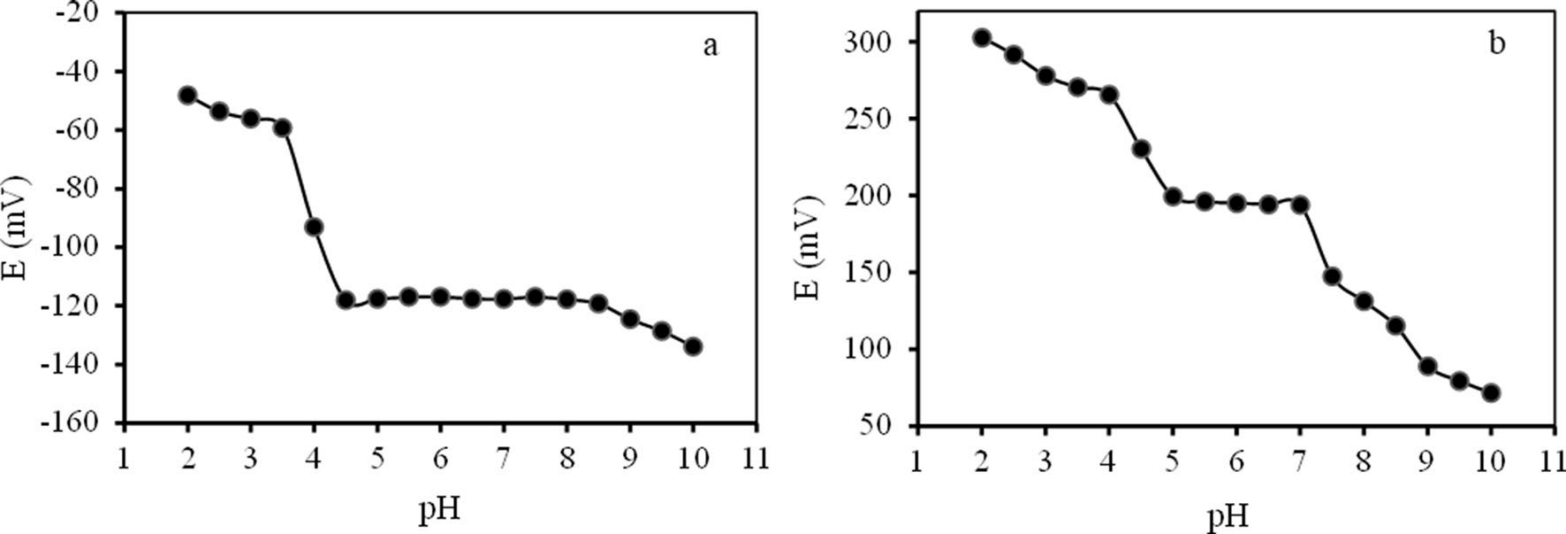
The effects of pH on the potential response of the (**a**) copper (II) selective CPE and (**b**) chromium (III) selective CPE.

The test solution’s pH is one of the most important factors in most ISEs’ performance. The effect of the solution’s pH on the response of the proposed modified electrodes was assessed on 1.0 × 10^–4^ mol L^−1^ analyte ions with various pH values. Copper (II) and chromium (III) solutions were prepared with different pH in the range of 2 to 10 by adding dilute solutions of NaOH or HNO_3_. Figure 2 shows the potential variation as a function of pH. During all experiments, the electrode’s composition was maintained constant. According to the results, the potential is constant in the pH range of 4.5 to 8.5 (Fig. 2a) and 5–7 (Fig. 2b) for modified copper (II) and chromium (III) selective electrodes, respectively. These results suggest the good performance of the sensors in this pH range. The fluctuations below pH of 4.5 and 5 could be associated with the partial protonation of the utilized ionophore. Protons can compete with copper (II) and chromium (III) at higher H^+^ concentrations. Based on the observations, at pH values below 4.5 and 5, the concentration of H^+^ is higher enough to win in the competition with copper (II) and chromium (III), respectively. At higher pH values, the changes could be caused by the formation of various copper (II) and chromium (III) hydroxides^26,28^.

### The response time of modified CPE

**Figure 3:**
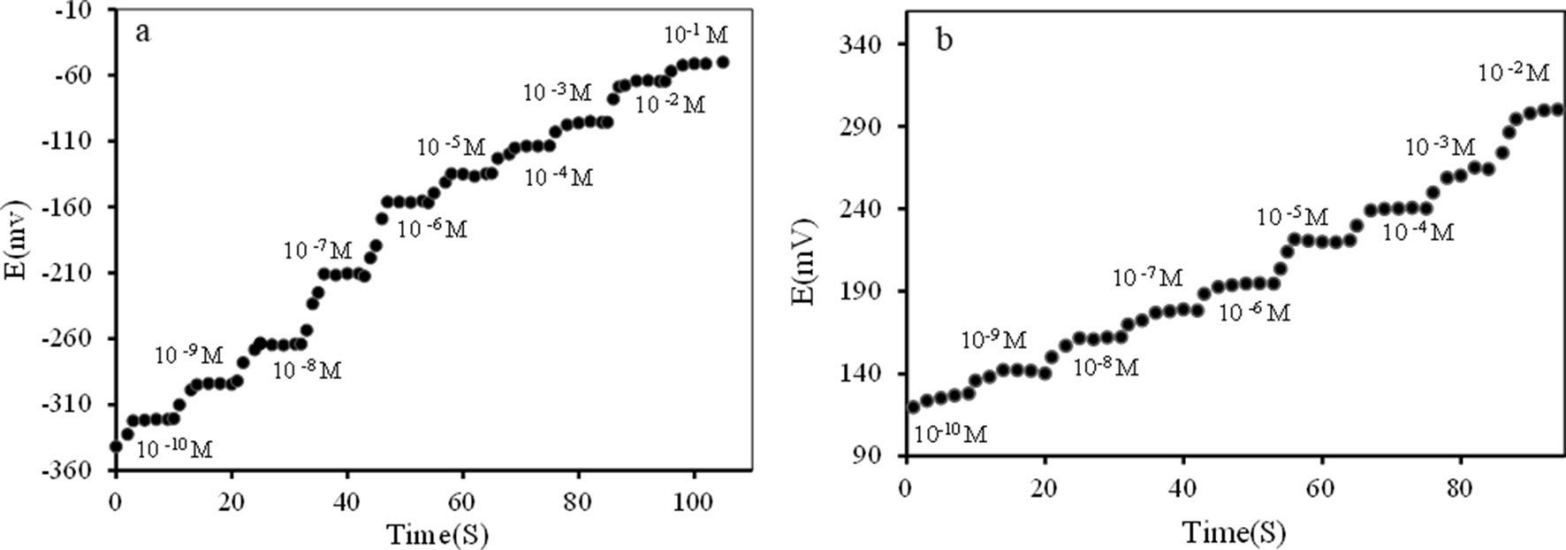
Response time of (**a**) copper (II) selective CPE and (**b**) chromium (III) selective CPE in various concentrations of ions.

Response time is the time required for the potential response to obtain values within ± 1 mV of the ultimate equilibrium potential^18^. It is a key parameter to detect the application of the suggested technique in routine works. The average response time was about 3–4 s in low to high concentrations in the case of the copper (II) selective CPE (Fig. 3a), and in the case of the chromium (III) selective CPE, it was about 3–5 s (Fig. 3b). The quick exchange kinetics of complexation-decomplexation of copper (II) and chromium (III) ions with the ionophores at the boundary of carbon paste-solution can be the reason for these short response times.

### Interference study

The response of an ion-selective electrode to the target ion is one of its key features over other ions and species existing in the solution. This feature is termed the potentiometric-selectivity coefficient.

**Figure 4:**
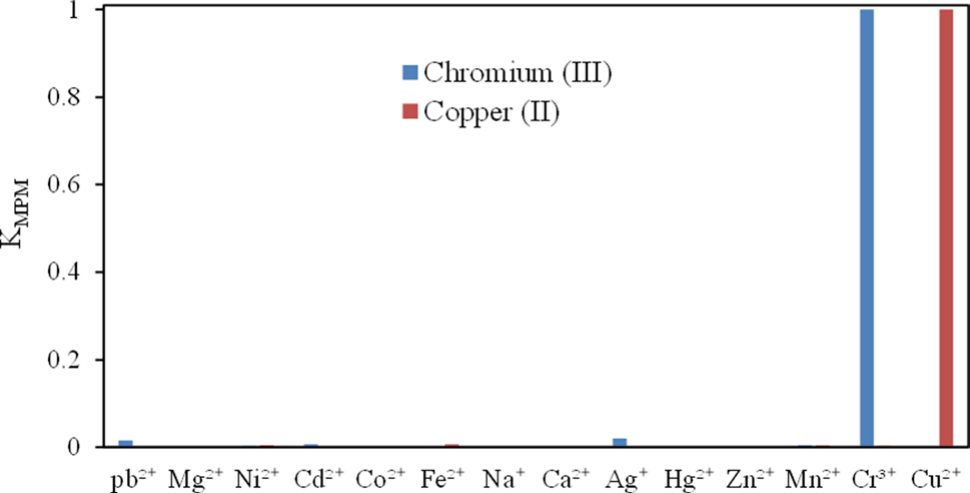
The values of K_MPM_ of proposed CPEs for a variety of different cations; red bars represent K_MPM_ of copper (II) selective electrode, and blue bars represent K_MPM_ chromium (III) selective electrode.

The selectivity coefficients of the two suggested electrodes were assessed by the well-known Matched Potential Method (MPM)^34,35^. In MPM, first, the changes in the potentials by altering the primary ion activity are determined. The interfering ion is then added to an equal reference solution until achieving the same potential changes. The selectivity coefficient K_MPM_ is obtained as:

$${\text{K}}_{{{\text{MPM}}}} = \, \Delta {\text{a}}_{{\text{A}}} /{\text{a}}_{{\text{B}}}$$

where Δa_A_ = a′_A_–a_A_, a_A_ represents the initial primary ion activity and a′_A_ the activity of A, while existing the interfering ion (i.e., a_B_). A K_MPM_ value of 1.0 represents the sensor with similar responses to both interfering and primary ions, with the smaller values denoting its higher selectivity. The results obtained are summarized in Table 2 and Fig. 4. All selectivity coefficients are much less than 1.0, indicating the higher selectivity of the developed electrodes for the analytes.

**Table 2:** The selectivity coefficients of different interfering cations for copper (II) and chromium (III) selective CPEs.

Ion	K_MPM_	
	Copper (II) selective CPE	Chromium (III) selective CPE
Pb^2+^	1.58 × 10^–5^	1.58 × 10^–2^
Mg^2+^	3.16 × 10^–4^	5.01 × 10^–4^
Ni^2+^	5.01 × 10^–3^	2.51 × 10^–3^
Cd^2+^	3.98 × 10^–4^	6.31 × 10^–3^
Co^2+^	3.98 × 10^–5^	1.99 × 10^–4^
Fe^3+^	6.30 × 10^–3^	5.01 × 10^–4^
Na^+^	3.96 × 10^–5^	6.31 × 10^–4^
Ca^2+^	1.99 × 10^–3^	3.16 × 10^–3^
Ag^+^	1.25 × 10^–5^	1.99 × 10^–2^
Hg^2+^	3.89 × 10^–5^	1.26 × 10^–5^
Zn^2+^	1.25 × 10^–3^	3.16 × 10^–4^
Mn^2+^	3.98 × 10^–3^	5.01 × 10^–4^
Cr^3+^	2.51 × 10^–3^	1
Cu^2+^	1	3.16 × 10^–4^

### Analytical characteristics of the modified CPEs

In this step, measuring ranges, detection limits (DL), and repeatability of the modified CPEs were evaluated.

**Figure 5:**
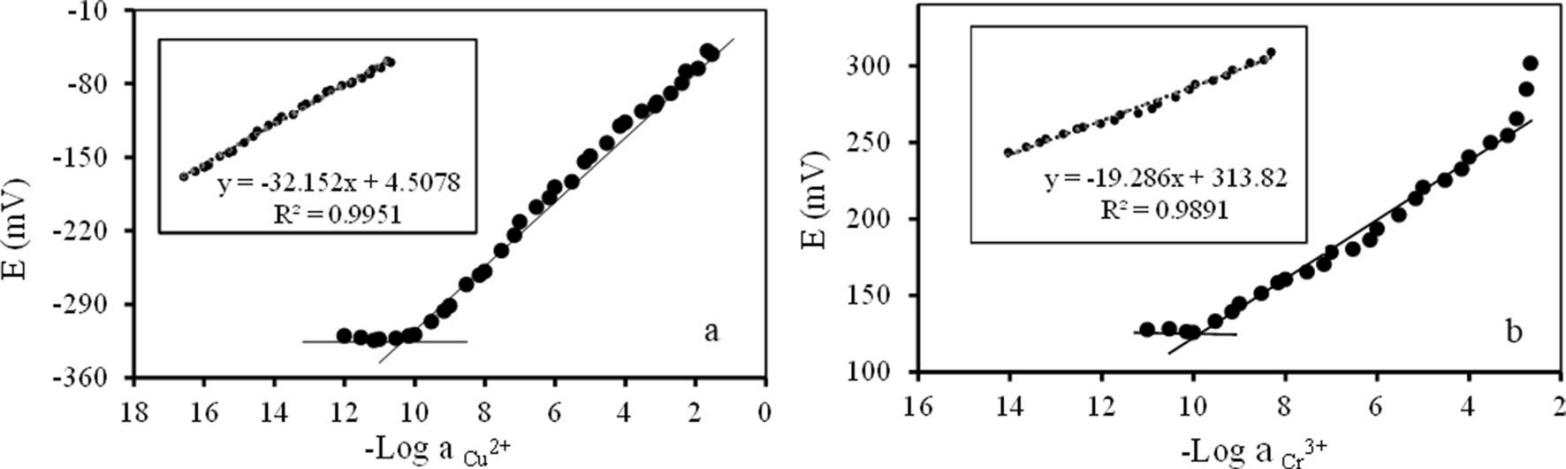
Calibrations curves of (**a**) copper (II) and (**b**) chromium (III) selective CPEs.

The potential response of electrodes showed a linear concentration (activity) range of 1 × 10^–1^ to 1 × 10^–10^ mol/L and 7 × 10^–3^ to 1 × 10^–10^ mol/L of copper (II) and chromium (III) ion in the calibration solution with Nernstian slopes of 32.15 and 19.28 mV/decade (Fig. 5) for copper (II) and chromium (III) selective CPEs, respectively. The Nernstian slope is a very interesting factor in discovering whether ion-selective electrodes are conveniently employed in analytical applications. This value is estimated using the slope curve of the potential measured in (EMF/mV) against the log activity of the standard solution (M) (Fig. 5). The optimal value of 59.10/n (mV/decade) was obtained for the Nernstian slope, where (n) is the valency^9^. Since the Nernstian slopes in this research were 32.15 and 19.28 mV/decade for copper (II) and chromium (III) selective CPEs, respectively, the proposed electrodes are acceptable to use in the analytical applications of copper (II) and chromium (III).

The detection limits were calculated by substituting the potential value that is the projection of the cut-off point in the correct equation. The electrodes display a low detection limit of 9.0 × 10^–11^ mol/L and 1.0 × 10^–10^ mol/L for copper (II) and chromium (III), respectively.

The repeatability of the electrodes was assessed with 4 repetitive measurements of analyte solution (1.0 × 10^–6^ mol L^−1^) under the optimized circumstances with modified CPEs. The precision of ion measurement by copper (II) and chromium (III) selective CPEs (based on the relative standard deviation) were determined to be 4.8% and 4.3%, respectively.

### Stability and lifetime of the CPEs

**Figure 6:**
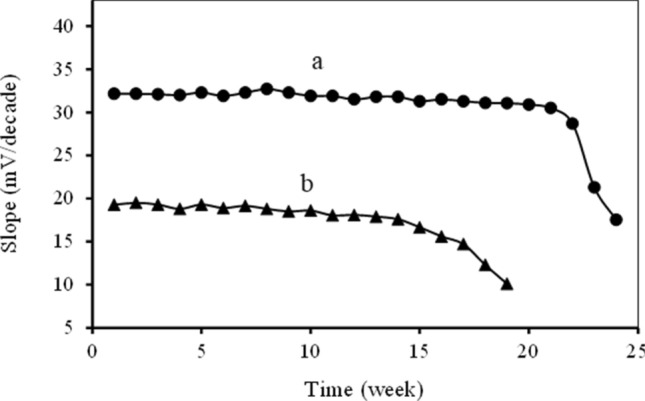
The lifetime of the (**a**) copper (II) and (**b**) chromium (III) selective CPEs.

The long terms stability and lifetime of the proposed CPEs were assessed by recalibration in standard solutions periodically and calculation of the optimized electrode’s slope within some weeks in the linear concentration range of each of the electrodes (Fig. 6). During this period, the CPEs were used weekly. The suggested electrodes were washed gently with distilled water, dried, and kept at room temperature.

The obtained results revealed that the copper (II) selective CPE could be used for 21 weeks with no considerable changes in its slope. In the case of chromium (III) selective CPE, the lifetime was about 13 weeks. By the end of this time, there was a slight gradual reduction in the slopes, indicating that the lifetime of the presented copper (II) and chromium (III) selective CPEs were more than 5 and 3 months, respectively.

### Sensing and selectivity mechanism

Based on the general principle of the working mechanism of the potentiometric ion-selective electrodes, during a potentiometric test, the ion recognition element (known as ionophore) located at the electrode surface interacts with the target ion and up-takes it to the electrode surface. Thus, the considered ionic species’ electrochemical potential on the electrode surface is increased compared with the solution layer adjacent to the electrode. Hence, an electrical potential is created across the electrode/solution interface.

Herein, the ligand A and ligand B play the role of ionophore for the CPEs. Due to the existence of oxygen and nitrogen electron-donating atoms in the structure of these ionophores, charge/dipole interactions and their complexation with transition and soft metals are predictable.

In the 1960s, Ralph Pearson classified Lewis bases and Lewis acids as soft and hard. The acids and bases suitable for interaction can be predicted using Pearson’s hard and soft (Lewis) acid–base (HSAB). It is noteworthy that metal ions are electron-pair acceptors and thus are Lewis acids, while ligands are electron-pair donors and thus are Lewis bases. Therefore, the Lewis acid–base theory can be used to describe metal–ligand interactions. As copper (II) ions are a soft acid in Pearson classification, they are expected to have stronger interactions with the relatively soft nitrogen atoms of ligand A as a Lewis base^36^.

But, in the case of the ligand B, adding a nitro group to the phenyl ring affected the ionophore selectivity. Here, the selectivity has changed from copper (II) to chromium (III). It seems that due to the resonant and electron-withdrawing effect of the nitro group, the hardness of the amide’s nitrogen atom increases and its affinity to harder ions such as chromium (III) increases. In other words, its tendency to share its electrons in a covalent bond has decreased and, therefore, it needs a more electronegative and stronger acid such as chromium (III) to form a complex. Table 3 compares the absolute electronegativity and hardness of the copper (II) and chromium (III) ions.

**Table 3:** Absolute electronegativity and hardness of the copper (II) and chromium (III) ions.

Ion	Absolute electronegativity (eV)	Hardness (eV)
Cu^2+^	28.56	8.27
Cr^3+^	40.0	9.1

### Analytical applications

The applicability of the suggested CPEs was evaluated by using them as indicator electrodes in the titration of 25 mL 1.0 × 10^–4^ mol/L copper (II) and chromium (III) solution with 1.0 × 10^–2^ mol/L EDTA.

**Figure 7:**
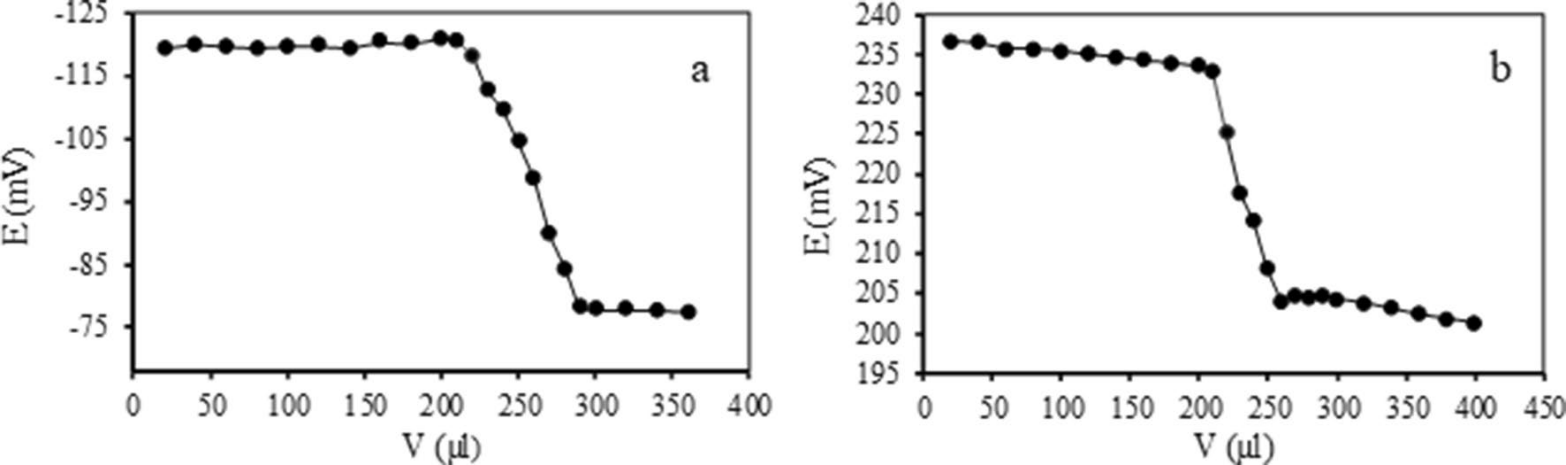
The potential titration curves of 25 mL 1.0 × 10^–4^ mol/L analyte solution with 1.0 × 10^–2^ mol/L of EDTA by using (**a**) copper (II) and (**b**) chromium (III) selective CPEs.

The resultant curves (Fig. 7) indicate that increasing the amount of EDTA leads to a reduction in the potential values because of creating complexation between analyte ions with EDTA and decreasing the concentration of free analyte ions in solution. The sharp endpoint in titration curves causes the accurate estimation of copper (II) and chromium (III) concentration throughout the experiment.

For examining the applicability of the two proposed electrodes to real specimens, the enhanced electrodes were used to determine copper (II) and chromium (III) amounts in industrial manufactory wastewater samples. The samples were diluted 10 times and the pH values of the solutions were set. The analysis was performed by the proposed CPEs and flame atomic absorption spectroscopy (FAAS) methods. The results of the measurements are shown in Table 4. As can be observed, the results were comparable with those obtained by FAAS. Thus, both modified CPEs can be a good alternative to determine successfully copper (II) and chromium (III) in real samples.

**Table 4:** Analysis of copper (II) and chromium (III) in wastewater samples.

Samples	Measured by copper (II) selective CPE	Copper (II) Measured by FAAS	Measured by chromium (III) selective CPE	Chromium (III) Measured by FAAS
Wastewater	6.6 × 10^–4^ (± 4.5) M	6.7 × 10^–4^ (± 2.1) M	1.8 × 10^–2^ (± 4.1) M	1.5 × 10^–2^ (± 2.4) M

Also, the chromium (III) selective CPE was used to specify hexavalent and trivalent chromium in standard samples. As shown in Table 5, the results obtained by the suggested CPE and the real amount of the hexavalent and trivalent chromium are in satisfactory agreement.

**Table 5:** Determining the trivalent and hexavalent chromium in standard sample.

Ion	Actual concentration	Measured concentration
Cr^3+^	3.8 × 10^–3^ M	4.0 × 10^–3^ M
Cr^6+^	1.9 × 10^–3^ M	1.7 × 10^–3^ M

### Comparison study

Table 6 compares some important features of the presented CPEs with the equivalent values for some formerly reported copper (II) and chromium (III) selective electrodes in terms of different ionophores^37–48^.

**Table 6:** Comparison between the suggested CPEs and the formerly similar reported copper (II) and chromium (III) selective electrodes in literature.

Ion	Ligand	Working range (M)	DL* (M)	Slope (mV decade^−1^)	pH	R.T** (s)	L.T*** (month)	Ref
Cu^2+^	3,4-dihydro-4,4,6-trimethyl-2(1H)-pyrimidine thione	9.77 × 10^–7^–7.6 × 10^–2^	7 × 10^–7^	30	2	45	2	^37^
Cu^2+^	1-(3-imino-4-hydroxophenylazo-4 nitrobenzene)	3.5 × 10^–9^–1 × 10^–2^	1.44 × 10^–9^	–	–	–	–	^38^
Cu^2+^	N,N’-bis(3- methylsalicylidene)-p-diphenylene methane diamine	1 × 10^–6^–1 × 10^–1^	–	30.1	2–6.5	–	–	^39^
Cu^2+^	bis [o-(N-methylidenamino-2-thiol-1,3,4-thiadiazole-5-yl) phenoxy] ethane	4 × 10^–9^–2.2 × 10^–3^	1.6 × 10^–9^	29.3	4–9	15	–	^40^
Cu^2+^	5,10,15,20-tetrakis(4-hydroxyphenyl) porphyrin	4 × 10^–9^–2 × 10^–1^	2 × 10^–9^	30.1	–	5	2	^41^
Cu^2+^	MWCNTs	10^–6^–10^–2^	1.1 × 10^–6^	28.4	–	1	–	^42^
Cu^2+^	Etioporphyrin	1.28 × 10^–6^–1.28 × 10^–2^	8.99 × 10^–7^	30.3	4.5–8.5	5	–	^43^
Cu^2+^	4-methylcoumarin-7-yloxy-N-phenyl acetamide	1 × 10^–10^–1 × 10^–1^	9 × 10–11	32.15	4.5–8.5	3–4	5	This work
Cr^3+^	2-acetylpyridine & nanoporous silica gel	1 × 10^–8^–1 × 10^–3^	8 × 10^–9^	19.8	1.5–5	55	–	^44^
Cr^3+^	Chitosan schiff base derivative	1 × 10^–7^–1 × 10^–2^	5.6 × 10^–8^	19.5	5.5–9	5–10	2	^45^
Cr^3+^	(N1)3-methylpyrazol-5-one	(N1)1 × 10^–6^–1 × 10^–2^	–	19.8	5–9	10	2	^46^
Cr^3+^	(N2) MWCNT-CoCl	(N2)1 × 10^–7^–1 × 10^–2^	–	19.7	3–9	15	2	^46^
Cr^3+^	1,3-Bis[4-amino-5-benzyl-1,2,4-triazol-3-ylsulfanyl]propane	1 × 10^–8^–5 × 10^–2^	8 × 10^–9^	20.0	2.3–5.2	10	3	^47^
Cr^3+^	4-((E)-(2-amino-4 chlorophenylimino) methyl)-5-(hydroxymethyl)-2-methylpyridin-3-ol	1 × 10^–10^–1 × 10^–2^	–	–	3.5–9	10	2	^48^
Cr^3+^	4-methylcoumarin-7-yloxy-N-4-nitrophenyl acetamide	1 × 10^–10^–7 × 10^–3^	1 × 10^–10^	19.28	5–7	3–5	3	This work

*Detection Limit, ** Response Time, *** Life Time.

According to the table, in most cases, the suggested electrodes represent superior performance to the formerly reported electrodes. Overall, the results show an improvement in the working range, detection limit, response time, usable pH range, and the lifetime of copper (II) selective CPE. Regarding chromium (III) selective CPE, an improvement in the working range, detection limit, and response time can be seen, and the electrode lifetime is similar to the best-reported chromium (III) selective CPEs.

## Experimental

### Reagents and materials

Ionophores were synthesized using aniline, 4-nitroaniline, resorcinol, ethyl acetoacetate, sulfuric acid, ethyl bromoacetate, potassium carbonate, acetone, hydrazine hydrate, ethanol, tetrahydrofuran (THF), and copper chloride purchased from Sigma-Aldrich. Graphite powder with particle size < 20 μm from Fluka and high-purity paraffin oil and multi-walled carbon nanotubes from Sigma-Aldrich were used to prepare carbon pastes. Potassium dichromate, sodium thiosulfate, and nitrate salts of the used cations (from Merck and Sigma-Aldrich) were of the highest available purity. Double distilled deionized water was used during the experiment.

### Apparatus

Fourier transform infrared spectra were recorded on a Bruker Tensor 27 instrument using the KBr disks. ^1^H and ^13^C nuclear magnetic resonance (NMR) spectra were recorded with an Ultra shield Bruker 400 instrument using CDCl_3_ as the deuterated solvent. A Bransetead Electro-Thermal B1 tool was used to determine the melting point. UV–vis spectrophotometer (Varian Cary100-Bio) was utilized to achieve absorbance curves and absorbance spectra.

The constructed ion-selective CPE, as the working electrode, was placed in a glass cell along with a double junction Ag/AgCl as the reference electrode for potentiometric measurement. The two electrodes were linked to a digital milli-voltmeter (HIOKI 3256.50) with ± 0.1 mV precision. The pH controlling was performed using a Metrohm pH-meter with a combined glass electrode.

### Synthesis of ionophores

**Figure 8:**
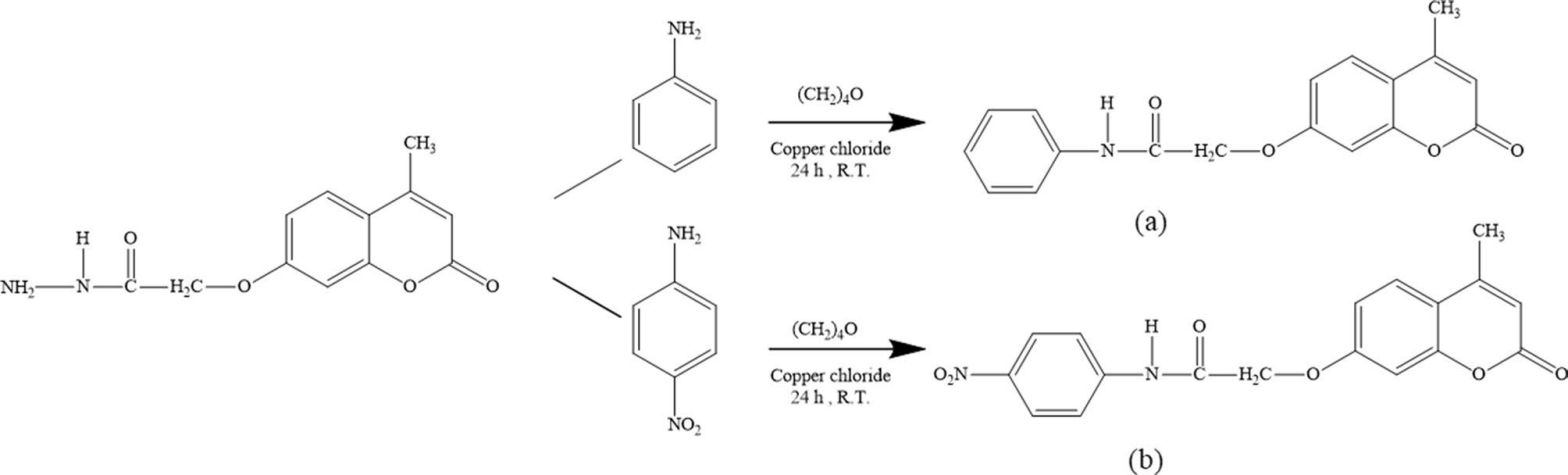
Synthesis of (**a**) 4-methylcoumarin-7-yloxy-N-phenyl acetamide and (**b**) 4-methylcoumarin-7-yloxy-N-4-nitrophenyl acetamide.

First, 4-methyl-7-hydroxy coumarin, 4-methylcoumarin-7-yloxy ethyl acetate, and 4-methylcoumarin-7-yl-oxy acetohydrazide were synthesized as described in previous reports^49,50^. Then, for the synthesis of the ligand A (Fig. 8a) and ligand B (Fig. 8b), two 100 mL round-bottomed flasks were used as reaction vessels and equipped with a magnet bar and magnetic stirrer. About 10 mL of tetrahydrofuran was used as the solvent. Next, 0.1 g of 4-methylcoumarin-7-yl-oxy acetohydrazide, 0.34 g of copper (II) chloride, and 0.21 g of aniline or 4-Nitroaniline were added to the reaction vessels (Fig. 8). The reaction mixture was stirred at room temperature for 24 h.

The advancement of the reactions was monitored using thin-layer chromatography (TLC) on silica-gel as the stationary phase, and CHCl_3_-MeOH (2:1) was used as the mobile phase. After completing the reactions, the products were separated from the reaction mixture by liquid extraction and recrystallized using appropriate solvents. The melting point of ligand A and B were determined to be 228℃ and 274℃, respectively.

Also, the structures were characterized by H-NMR, FT IR, ^1^ and ^13^C-NMR spectroscopy techniques as follows:

Ligand A (4-methylcoumarin-7-yloxy-N-phenyl acetamide): IR (KBr, cm^−1^) 3364; 3075; 3040; 2915; 1703; 1660; 1625.5; 1539; 1500; 1444; 1363; 1297; 1079; 1151; 1209; 887; 850; 801; 752; 696. 1HNMR: (DMSO-d6) δ, ppm, 2.38(3H, s); 4.8(2H, s); 6.21(1H, s); 7–7.72(8H, m); 10.14(1H, s).

Ligand B (4-methylcoumarin-7-yloxy-N-4-nitrophenyl acetamide): IR (KBr, cm^−1^) 3303.4; 3000–3100; 291; 1689.3; 1617.4; 1566.5; 1501.3; 1395.1; 1346.6; 1332.3; 1287.4; 1261.6; 1195.8; 1164.4; 1140.2; 1111.7; 1088.5; 850.4; 831.4; 680. 1HNMR: (DMSO-d6) δ, ppm, 2.26(3H, s); 4.93(2H, s); 6.23(1H, s); 7.03(1H, s)-7.06(1H, s); 7.7(1H, d) 7.9(2H, d); 8.2(2H, d); 10.77(1H, s).

The ligand A and 4B were utilized as ionophores in the preparation of the copper and chromium selective carbon paste electrodes, respectively.

### Evaluation of the ionophore selectivity by UV–visible spectrophotometry

UV–visible spectrophotometry was used to assess the interaction of ionophores with some metal cations as follows. About 300 µL of aliquots of various cations solutions (1 mM) were added to a solution of ligand A and B at a concentration of 1.0 × 10^–5^ mol/L in acetonitrile and tetrahydrofuran. Next, the alterations in the ligand’s spectrum were measured.

### Electrode preparation

For fabricating the modified CPEs, the pastes were prepared by hand-mixing different amounts of the synthesized ionophore powder and various amounts of graphite, MWCNTs, and paraffin oil for 20 min. When each mixture was homogenized, a portion of the prepared paste was carefully packed into the tube tip to prevent possible air gaps. It regularly improves the electrode resistance. Electrical contact was established by inserting a copper wire into the CPE opposite end. Before usage, a piece of soft paper was used to smooth the carbon paste’s external surface. The old surface was scraped out, and the new carbon paste was replaced to make a new surface. The electrode was ultimately conditioned for 24 h by soaking in a 1.0 × 10^–3^ mol L^−1^ of analyte solution. The sensor’s response was validated by measuring the electromotive force (EMF) of the following electrochemical cell:

$${\text{Developedcarbon paste electrode}}\left| {\text{ sample solution }} \right|{\text{ Ag}}{-}{\text{AgCl }}\left( {\text{reference electrode}} \right)$$

For potential measurement at 25 ± 0.1 ℃, the explained reference electrode and the carbon paste electrode were linked to a digital milli-voltmeter. The plot of EMF was made as a function of log [analyte].

### Speciation of trivalent and hexavalent chromium in standard sample

To determine the concentration of Cr (VI), it must be converted to Cr (III). Therefore, before determining the Cr (VI), adding 0.1 mL of sodium thiosulfate (0.1 mol L^-1^) to the solution is essential. First, Cr (III) was defined, followed by adding sodium thiosulfate to the solution and reducing Cr (VI) to Cr (III). Next, it was determined as total chromium; the difference denotes the presence of Cr (VI) in the sample^4^.

## Conclusion

Two new modified carbon paste electrodes were fabricated for the selective and sensitive detection of copper (II) and chromium (III) using two similar derivatives of the 4-methylcoumarin. To this end, 4-methylcoumarin-7-yloxy-N-phenyl acetamide (ligand A) and 4-methylcoumarin-7-yloxy-N-4-nitrophenyl acetamide (ligand B) were synthesized and used as a new neutral carrier to design CPEs. It was found that copper (II) and chromium (III) recognition of these modified CPEs were highly affected by the nature and structure of the ionophores. Also, ligand A showed good copper (II) ions selectively, while ligand B tended to chromium (III). Due to the resonant and electron-withdrawing effect of the nitro group in ligand B, the hardness of the amide’s nitrogen atom increases compared to the ligand A. Therefore, its affinity to harder ions such as chromium (III) increases. The tendency of ionophores to copper (II) and chromium (III) ions was confirmed by UV–visible spectrophotometry. According to the obtained results, two novel potentiometric modified carbon paste electrodes were fabricated in the case of the copper electrode by 5% ionophore, 65% graphite powder, 5% MWCNT, and 25% paraffin oil. In the case of chromium electrode, these conditions were 20% ionophore, 50% graphite powder, 5% MWCNT, and 25% paraffin oil. Also, the results showed that the MWCNTs could be used as a superior candidate to modify CPE due to the excellent electrical conductivity and large specific surface area. The copper (II) selective CPE showed a Nernstian slope of 32.15 mV/decade over the concentration range of 1.0 × 10^–10^–1.0 × 10^–1^ mol L^−1^, while chromium (III) selective CPE showed a Nernstian slope of 19.28 mV/decade over the concentration range of 1.0 × 10^–10^–7.0 × 10^–3^ mol L^−1^. The electrodes indicate a stable and short response time of about 3–5 s. The proposed modified CPEs were used successfully to determine copper (II) in wastewater samples and for the determination and speciation of chromium (III) and chromium (VI). The modified CPEs demonstrated the benefits of low cost, simple design, excellent selectivity to analytes, wide concentration range, renewability, and applicability as an indicator electrode.

## Data Availability

The datasets generated during and/or analysed during the current study are available from the corresponding author on reasonable request.

## References

[1] Ozdemir S, Kilinc E, Oner ET (2019). Preconcentrations and determinations of copper, nickel and lead in baby food samples employing *Coprinus silvaticus* immobilized multi-walled carbon nanotube as solid phase sorbent. Food Chem..

[2] Song Y, Yang LY, Wang YG, Yu D, Shen J, Ouyang XK (2019). Highly efficient adsorption of Pb(II) from aqueous solution using amino-functionalized SBA-15/calcium alginate microspheres as adsorbent. Int. J. Biol. Macromol..

[3] Hajiaghababaei L, Amini Z, Shahvelayati AS (2018). Removal of Pb^2+^, Cu^2+^ and Ag^+^ cations from wastewater by modified ZnO nanoparticles with S,N-substituted thiouracil derivative. J. Elem..

[4] Ganjali MR, Hajiagha Babaei L, Badiei A, Saberian K, Behbahani S, Mohammadi Ziarani G, Salavati-Niasari M (2006). A novel method for fast enrichment and monitoring of hexavalent and trivalent chromium at the ppt level with modified silica MCM-41 and its determivation by inductively coupled plasma optical emission spectrometry. Quim. Nova..

[5] Nusko R, Heumann KG (1996). Cr(III)/Cr(VI) speciation in aerosol particles by extractive separation and thermal ionization isotope dilution mass spectrometry. Fresenius. J. Anal. Chem..

[6] Shemirani F, Abkenar SD, Mirroshandel AA, Niasari MS, Kozania RR (2003). Preconcentration and speciation of chromium in water samples by stomic absorption spectrometry after cloud-point extraction. J. Anal. Sci..

[7] Bagherian G, Chamjangali M, Evari H, Ashrafi M (2019). Determination of copper(II) by flame atomic absorption spectrometry after its perconcentration by a highly selective and environmentally friendly dispersive liquid–liquid microextraction technique. J. Anal. Sci. Technol..

[8] Elyas Sodan N, Elci SG, Arslan Kartal A, Hol A, Elci L (2021). Speciation and preconcentration of chromium in real samples by magnetic polythiophene nanoparticle solid-phase extraction (SPE) coupled with microsampling injection–flame atomic absorption spectrometry (FAAS). Instrum. Sci. Technol..

[9] Shishov A, Terno P, Bulatov A (2021). Deep eutectic solvent decomposition-based microextraction for chromium determination in aqueous environments by atomic absorption spectrometry with electrothermal atomization. Analyst.

[10] Shi J, Jiang Y, Zhang B, Zhou J (2021). Determination of Mn, Fe, Ni in copper alloy by X-ray fluorescence analysis. OALib..

[11] Rajib A, Tariqur Rahman M, Md. Ismail AB (2021). Using X-ray fluorescence technique propagation of chromium (Cr) from poultry feeds to different parts of chicken including her eggs. J. Phys. Conf. Ser..

[12] Chernikova I.I., F. S. S., Ermolaeva T.N. Analysis of copper alloys by inductively coupled plasma atomic emission spectrometry with spark sampling. *Ind. Lab. Diagn. Mater*. **86**, 11–1. 10.26896/1028-6861-2020-86-3-11-19 (2020).

[13] Tsanaktsidou E, Zachariadis G (2020). Titanium and chromium determination in feedstuffs using ICP-AES technique. J. Sep..

[14] Manousi N, Zachariadis GA (2020). Development and application of an ICP-AES method for the determination of nutrient and toxic elements in savory snack products after autoclave dissolution. J. Sep..

[15] Hajiaghababaei L, Shahvelayati AS, Aghili SA (2015). Rapid determination of cadmium: A potentiometric membrane sensor based on ninhydrin-pyrogallol monoadduct as a new ionophore. Anal. Bioanal. Electrochem..

[16] Zhang L, Wei Z, Liu P, Wei H, Ma D (2021). Performance comparison of solid lead ion electrodes with different carbon-based nanomaterials as electron-ion exchangers. J. Sens..

[17] Panahi Sarmad A, Hajiaghababaei L, Shahvelayati AS, Najafpour J (2021). Development of copper-selective potentiometric sensor using a new ion carrier: A theoretical and experimental study. Russ. J. Electrochem..

[18] Hajiaghababaei L, Sharafi A, Suzangarzadeh S, Faridbod F (2013). Mercury recognition: A potentiometric membrane sensor based on 4-(benzylidene amino)-3,4-dihydro-6- methyl-3-thioxo-1,2,4-triazin-5(2H)one. Anal. Bioanal. Electrochem..

[19] Faridbod F (2016). Pramipexole symmetric and asymmetric potentiometric PVC membrane sensors. Int. J. Electrochem. Sci..

[20] Hassan SSM, Kamel AH, Amr AEGE, Fathy MA, Al Omar MA (2020). Paper strip and ceramic potentiometric platforms modified with nano-sized polyaniline (PANi) for static and hydrodynamic monitoring of chromium in industrial samples. Molecules.

[21] Rashvand  HR, Hajiaghababaei L, Darvich MR, Jalali Sarvestani MR, Jaberi Miyandoab F (2020). A liquid membrane mercury selective electrode based on 2-(NPipyridino methyl)-1-cyano cyclohexanol as a novel neutral carrier. J. Anal. Chem..

[22] Jafari M, Hajiaghababaei L, Darvish MR (2019). Oxime-2-(1-cyclohexenyl) cyclohexanone: synthesis and application as a new ion carrier for preparation of cobalt selective potentiometric electrode. Anal. Bioanal. Electrochem..

[23] Heydari Z, Hajiaghababaei L, Darvish MR (2019). Rapid measuring of Cu^2+^ ions by selective potentiometric sensor based on a newiIon carrier. Anal. Bioanal. Electrochem..

[24] Khalil S (2021). Chitosan based nano-membrane for chromium(III) determination in pharmaceutical and foodstuff samples. Int. J. Electrochem. Sci..

[25] Ali TA, Mohamed GG (2021). Development of chromium(III) selective potentiometric sensors for its determination in petroleum water samples using synthesized nano Schiff base complex as an ionophore. J. AOAC Int..

[26] Frag EY, Mohamed NM, Elashery SEA (2021). Exploitation of o-benzoyl benzoic acid as an efficient electroactive material for selective determination of Cr (III) ions in pharmaceutical samples and industrial waste water using carbon sensor. Anal. Chim. Acta..

[27] Frag EY, Mohamed MEB, Fahim EM (2018). Application of carbon sensors for potentiometric determination of copper(II) in water and biological fluids of Wilson disease patients. Studying the surface reaction using SEM, EDX, IR and DFT. Biosens. Bioelectron..

[28] Mashhadizadeh MH, Khani H, Foroumadi A, Sagharichi P (2010). Comparative studies of mercapto thiadiazoles self-assembled on gold nanoparticle as ionophores for Cu(II) carbon paste sensors. Anal. Chim. Acta..

[29] Afkhami A, Shirzadmehr A, Madrakian T, Bagheri H (2015). New nano-composite potentiometric sensor composed of graphene nanosheets/thionine/molecular wire for nanomolar detection of silver ion in various real samples. Talanta.

[30] Alizadeh T, Sabzi R, Alizadeh H (2016). Synthesis of nano-sized cyanide ion-imprinted polymer via non covalent approach and its use for the fabrication of a CN selective carbon nanotube impregnated carbon paste electrode. Talanta.

[31] Alizadeh T, Jamshidi F (2015). Synthesis of nanosized sulfate-modified α-Fe_2_O_3_ and its use for the fabrication of all-solid-state carbon paste pH sensor. J. Solid State Electrochem..

[32] Abbastabar-Ahangar H, Shirzadmehr A, Marjani K, Khoshsafar H, Chaloosi M, Mohammadi L (2009). Ion-selective carbon paste electrode based on new tripodal ligand for determination of cadmium (II). J. Incl. Phenom. Macrocycl. Chem..

[33] Jalali sarvestani MR, Hajiaghababaei L, Najafpour J, Suzangarzadeh S (2018). 1-(6-choloroquinoxaline-2-yl) hydrazine as an excellent ionophore for preparation of a cobalt selective electrode and potentiometric measuring of vitamin B12 in pharmaceutical samples. Anal. Bioanal. Electrochem..

[34] Umezawa Y, Umezawa K, Sato H (1995). Selectivity coefficients for ion-selective electrodes: Recommended methods for reporting K A, B pot values (Technical Report). Pure Appl. Chem..

[35] Umezawa Y, Umezawa K, Bühlmann P, Hamada N, Aoki H, Nakanishi J, Sato M, Xiao KP, Nishimura Y (2002). Potentiometric selectivity coefficients of ion-selective electrodes. Part II. Inorganic anions (IUPAC Technical Report). Pure Appl. Chem..

[36] Pearson RG (1988). Absolute electronegativity and hardness: Application to inorganic chemistry. Inorg. Chem..

[37] Abbaspour A, Moosavi SMM (2002). Chemically modified carbon paste electrode for determination of copper(II) by potentiometric method. Talanta.

[38] Bagheri H, Shirzadmehr A, Rezaei M (2016). Determination of copper ions in foodstuff products with a newly modified potentiometric carbon paste electrode based on a novel nano-sensing layer. Ionics.

[39] Mazloum Ardakani M, Mandegari AA, Masoum S, Naeimi H (2012). Multiwall carbon nanotubes modified carbon paste electrode for determination of copper(II) by potentiometric and impedimetric methods. J. Nanostruct..

[40] Mashhadizadeh MH, Ramezani S, Ebrahimi S (2012). Potentiometric determination of nanomolar concentration of Cu (II) using a carbon paste electrode modified by a self-assembled mercapto compound on gold nanoparticles. Sens. Actuators B Chem..

[41] Rajabi HR, Zarezadeh A, Karimipour G (2017). Porphyrin based nano-sized imprinted polymer as an efficient modifier for the design of a potentiometric copper carbon paste electrode. RSC Adv..

[42] Soleimani M, Afshar M (2013). Potentiometric sensor for trace level analysis of copper based on carbon paste electrode modified with multi-walled carbon nanotubes. Int. J. Electrochem. Sci..

[43] Issa YM, Ibrahim H, Shehab OR (2012). New copper(II)-selective chemically modified carbon paste electrode based on etioporphyrin I dihydrobromide. J. Electroanal. Chem..

[44] Zhou W, Chai Y, Yuan R, Guo J, Wu X (2009). Organically nanoporous silica gel based on carbon paste electrode for potentiometric detection of trace Cr(III). Anal. Chim. Acta..

[45] Sharif Manesh S, Masrournia M, Beyram Abady A (2020). Determination of chromium(III) and magnesium(II) ions in pharmacological and real water samples using potentiometric sensors based on chitosan schiff base derivative as green and sensitive ionophore. Anal. Bioanal. Electrochem..

[46] Heidari Z, Masrounia M (2018). A novel modified carbon paste electrode for the determination of chromium(III) in water. J. Anal. Chem..

[47] Zayed MA, Abbas AA, Mahmoud WH, Ali AE, Mohamed GG (2020). Development and surface characterization of a bis(aminotriazoles) derivative based renewable carbon paste electrode for selective potentiometric determination of Cr(III) ion in real water samples. Microchem. J..

[48] Shojaei E, Masrournia M, Beyramabadi A, Behmadi H (2020). Design and fabrication of carbon paste electrode for determination of Cr(III) ion in real water samples using a new synthesis Schiff base as selective ionophore. Eurasian Chem. Commun..

[49] Guan AY, Liu C, Li M, Li ZN, Zhang MX, Zhang H (2011). Synthesis and bioactivity of novel coumarin derivatives. Nat. Prod. Commun..

[50] Hamdi N, Al-Ayed AS, Ben Said R, Fabienne A (2012). Synthesis and characterization of new thiazolidinones containing coumarin moieties and their antibacterial and antioxidant activities. Molecules.

